# Sex-specific prognostic effect of CD66b-positive tumor-infiltrating neutrophils (TANs) in gastric and esophageal adenocarcinoma

**DOI:** 10.1007/s10120-021-01197-2

**Published:** 2021-05-19

**Authors:** Alexander Quaas, Aylin Pamuk, Sebastian Klein, Jennifer Quantius, Jan Rehkaemper, Atakan G. Barutcu, Josef Rueschoff, Thomas Zander, Florian Gebauer, Axel Hillmer, Reinhard Buettner, Wolfgang Schroeder, Christiane J. Bruns, Heike Löser, Birgid Schoemig-Markiefka, Hakan Alakus

**Affiliations:** 1 Department of General, Visceral, Cancer and Transplantation Surgery, University Hospital Cologne, Kerpener Str. 62, 50937 Cologne, Germany; 2 Institute of Pathology, University Hospital Cologne, Cologne, Germany; 3 Department I of Internal Medicine, Center for Integrated Oncology Aachen Bonn Cologne Duesseldorf, Gastrointestinal Cancer Group Cologne GCGC, University of Cologne, Cologne, Germany; 4 Institute of Pathology, Nordhessen and Targos Molecular Pathology GmbH, Kassel, Germany

**Keywords:** Tumor microenvironment, Neutrophils, Gender, Esophageal neoplasms, Stomach neoplasms

## Abstract

**Background:**

Tumor-associated neutrophils (TANs) have recently been identified as a relevant component of the tumor microenvironment (TME) in solid tumors. Within the TME TANs mediate either tumor-promoting or tumor-inhibiting activities. So far, their prognostic relevance remains to be determined. This study aims to evaluate the prognostic relevance of TANs in different molecular subtypes of gastric and esophageal adenocarcinoma.

**Methods:**

We analyzed a total of 1118 Caucasian patients divided into gastric adenocarcinoma (*n* = 458) and esophageal adenocarcinoma cohort (*n* = 660) of primarily resected and neoadjuvant-treated individuals. The amount of CD66b + TANs in the tumor stroma was determined using quantitative image analysis and correlated to both molecular, as well as clinical data.

**Results:**

An accumulation of TANs in the tumor stroma of gastric carcinomas was associated to a significant favorable prognosis (*p* = 0.026). A subgroup analysis showed that this effect was primarily related to the molecular chromosomal instable subtype (CIN) of gastric carcinomas (*p* = 0.010). This was only observed in female patients (*p* = 0.014) but not in male patients (*p* = 0.315). The same sex-specific effect could be confirmed in adenocarcinomas of the esophagus (*p* = 0.027), as well as in female individuals after receiving neoadjuvant therapy (*p* = 0.034).

**Conclusions:**

Together, we show a sex-specific prognostic effect of TANs in gastric cancer within a Caucasian cohort. For the first time, we showed that this sex-specific prognostic effect of TANs can also be seen in esophageal cancer.

**Supplementary Information:**

The online version contains supplementary material available at 10.1007/s10120-021-01197-2.

## Introduction

Gastric adenocarcinomas are one of the most common malignant tumors worldwide. [1, 2] Recently, the TCGA consortium has proposed four subgroups, including the chromosomally instable subtype (CIN; 50%), microsatellite-instable subtype (MSI; 20%), the genomic stable (GS; 20%) and Epstein-Barr virus-associated subtype (EBV; 10%). [3].

Genomically, adenocarcinomas of the esophagus correspond almost exclusively to the CIN subtype of gastric carcinoma. [4–6] In contrast to gastric cancer, the incidence of esophageal cancer continues to rise in Northern Europe, mainly affecting male individuals (ratio male to female, 7–9:1) [7, 8].

It is known that the immunological context of malignant tumors may have both pro-tumorigenic, as well as anti-tumorigenic properties. The significance of understanding the immunological context of malignant tumors is elucidated by recent advances in reprogramming immune cells to induce antitumoral effects.

Meanwhile, several studies have proposed both tumor-promoting and tumor-inhibiting properties of TANs. [9, 10] Interestingly, there are relevant differences in the composition of the tumor microenvironment between Asian and Non-Asian (Caucasian) patients. [11, 12] Currently, the significance of TANs in esophageal adenocarcinomas remains elusive. In addition, the distribution of TANs among different molecular subtypes of gastric cancer, as well as in patients treated following neoadjuvant therapy remains unknown.

Therefore, we evaluated CD66b-positive TANs in both gastric and esophageal cancer tissue using quantitative image analysis on a large Caucasian patient population that was divided into a gastric carcinoma cohort and an esophageal adenocarcinoma cohort.

## Methods und materials

### First clinical cohort: the gastric carcinoma cohort (*n* = 458)

**Fig. 1:**
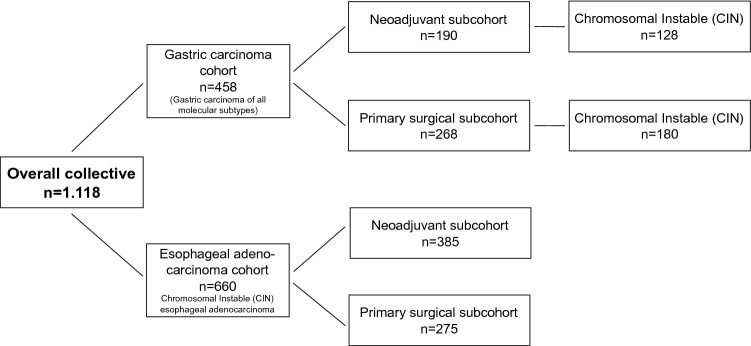
Illustration of the structure of the analyzed patient collectives categorized into gastric carcinoma and the esophageal adenocarcinoma. The gastric carcinomas were divided into the four molecular subgroups (according to TCGA). This revealed an accumulation and special prognostic relevance of the TANs in the CIN subgroup. Adenocarcinomas of the esophagus show the same molecular characteristics as the CIN subgroup of the stomach and are therefore particularly well suited as a “validation”. Both collectives were divided into two cohorts: primarily operated patient cohorts and neoadjuvant-treated patient cohorts. Nothing is known about the significance and distribution of TANs in the adenocarcinoma of the esophagus, the neoadjuvant-treated carcinomas nor in the different molecular subgroups of gastric carcinoma. *TCGA* The Cancer Genome Atlas

The gastric carcinoma cohort consisted of 458 tumors. These cases were divided into two sub-cohorts: (i) 268 patients had undergone primary surgery (ii) 190 patients who had received neoadjuvant therapy before surgery (Fig. 1; Table 1).

**Table 1:** Patient characteristics of the gastric adenocarcinoma including the four molecular subtypes (according to TCGA), separated into the overall collective, male and female cohort

	Overall collective (*n* = 458)		Male cohort (*n* = 310)		Female cohort (*n* = 148)	
Preoperative treatment						
None	268	58.5%	176	56.8%	92	62.2%
Neoadjuvant	190	41.5%	134	43.2%	56	37.8%
Age						
< 50	58	13.4%	33	11.1%	25	18.1%
> 50	376	86.6%	263	88.9%	113	81.9%
UICC stage						
(y)1	92	22.8%	63	22.9%	29	22.5%
(y)2	114	28.2%	74	26.9%	40	31.0%
(y)3	137	33.9%	97	35.3%	40	31.0%
(y)4	61	15.1%	41	14.9%	20	15.5%
Molecular subtype						
CIN	308	73.5%	208	74.3%	100	71.9%
MSI	37	8.8%	23	8.2%	14	10.1%
GS	55	13.1%	32	11.4%	23	16.5%
EBV	19	4.5%	17	6.1%	2	1.4%
Localisation						
Proximal	186	41.6%	142	47.3%	44	29.9%
Corpus	125	28.0%	68	22.7%	57	38.8%
Distal	99	22.1%	61	20.3%	38	25.9%
Other	37	8.3%	29	9.7%	8	5.4%

*TCGA* The Cancer Genome Atlas, *UICC* Union internationale contre le cancer, *CIN* chromosomal instability, *MSI* microsatellite instability, *GS* genomically stable, *EBV* Epstein–Barr virus-positive

In the neoadjuvant-treated subgroup, three different regimens were used (PFL, MAGIC, FLOT, considering patients over the last 20 years). The majority of the patients were treated according to MAGIC and FLOT protocols. Data regarding response to neoadjuvant chemotherapy was available in 119 patients (62.7%): > 50% vital tumors: 80 patients (67.2%), 10–50% vital tumors: 29 patients (24.4%), < 10% vital tumors: 9 patients (7.6%) and complete response: 1 patient (0.8%).

### Second clinical cohort: the esophageal adenocarcinoma cohort (*n* = 660)

The second cohort consisted of 660 adenocarcinomas of the esophagus, divided into (i) patients being primarily resected (*n* = 275) and (ii) neoadjuvant treated (*n* = 385) (patient’s characteristics, Table 5). The selection of this cohort was made after subgroup analyses of the gastric tumor cohort in which a prognostic relevance of chromosomal instable gastric carcinomas (CIN) was found. Since almost 100% of the adenocarcinomas of the esophagus also corresponded to the molecular CIN group, adenocarcinomas of the esophagus were used to validate the relevance found in the CIN subtype of gastric carcinoma (Fig. 1, Table 5).

### Molecular subtyping

Cases of gastric cancers were classified according to the TCGA classification (CIN, MSI, GS and EBV). [3] The current WHO classification of tumors 2019 [13] has proposed a diagnostic algorithm to reconstruct these subtypes effectively. We have applied this algorithm to the gastric carcinomas of our collective: (1) the EBV subtype using the specific RNA in-situ test “EBER” (ready to use by Leica, Germany). This test specifically labels EBV RNA on formalin-fixed and paraffin-embedded tumor material (2) the MSI subtype by examining all tumors with two internationally recommended immunohistochemical antibodies (MLH1 (clone: M1 by Ventana/Roche) and MSH2 (clone: G219-1129) both ready to use). This was done primarily at the tissue microarray (TMA) and in case of all negative results (= loss of protein in the tumor cell nuclei with positive staining reaction in the surrounding inflammatory cells or fibroblasts as on-slide positive control) or unclear results a renewed immunohistochemical analysis on large tumor areas in combination with a partner protein analysis (PMS2 clone: EPR 3947 and MSH6 clone: 44, both ready to use). Microsatellite status was exemplarily determined using an in-house polymerase chain reaction (PCR) protocol with primers for the Bethesda-Markers including either the mononucleotide markers BAT25 and BAT26 as well as the dinucleotide markers D5S346, D2S123, D17S250 or the dinucleotide markers D2S123, D17S250, D10S197, D18S58, D13S153 and the tetranucleotide marker MYCL1. For evaluation, PCR was followed by fragment length analysis on an ABI PRISM 3500 Genetic Analyzer (Applied Biosystems, Life Technologies, Darmstadt, Germany).

All immunohistochemically determined mismatch-repair deficient tumors (d-MMR) were also microsatellite-instable (MSI-H) 3) for the GS subtype: all tumors with signet-ring cell histology (= diffuse type according to Lauren) or immunohistochemical loss of E-cadherin and not MSI or EBV were assigned to this category. The CIN subtype corresponded to all tumors that could not be assigned to the clearly defined other subgroups (EBV, MSI, GS). These tumors typically showed the following characteristics: intestinal (glandular) morphology, TP53 alteration (which we determined by immunohistochemistry, clone DO-7, Dako) and more frequent ERBB2 alteration (which we determined by immunohistochemistry (clone: 4B5 by Roche ready to use and Fluorescence in-situ (FISH) for Her2/neu).

### Surgery for adenocarcinomas of the stomach

Standardized surgical treatment included subtotal distal or total gastrectomy with trans-hiatal resection of the distal esophagus in case of an adenocarcinoma of the esophago-gastric junction (Siewert 2), and a systematic D2 lymphadenectomy with the goal of complete resection (R0). Roux-en-Y jejunal loop with gastrojejunostomy was considered the method of choice in the reconstruction procedures.

### Surgery for adenocarcinomas of the esophagus

The standard surgical procedure was laparoscopic gastrolysis and right transthoracic en bloc esophagectomy including two-field lymphadenectomy of mediastinal and abdominal lymph nodes. Reconstruction was performed by high intrathoracic esophagogastrostomy as described previously [14]. Patients with advanced esophageal cancer (cT3, cNx, M0) received preoperative chemoradiation (5-FU, cisplatin, 40 Gy as treated in the area prior the CROSS trial) or chemotherapy alone.

### Follow-up

During the first 2 years, patients were followed up clinically in the hospital every 3 months. Afterwards, annual exams were carried out. Follow-up examinations included a detailed history, clinical evaluation, abdominal ultrasound, chest X-ray and additional diagnostic procedures as required. Follow-up data were available for all patients. There is a preponderance of minor responders in the TMAs, defined as histopathological residual tumor of ≥ 10%. [15].

### Tissue microarray (TMA)

For the gastric carcinoma cohort considering 458 patients and for the second cohort considering 660 patients with esophageal adenocarcinoma we created Tissue Microarrays using formalin-fixed, paraffin-embedded tumor material. For TMA one tissue core from each tumor sample was punched out and transferred into a TMA recipient block. TMA construction was performed as previously described [16, 17]. In brief, tissue cylinders with a diameter of 1.2 mm each were punched from selected tumor tissue blocks using a self-constructed semi-automated precision instrument and embedded in empty recipient paraffin blocks. To verify the staining procedure, positive and negative control tissues were punched out and added to the TMAs.

### Immunohistochemistry and quantitative image analysis

The CD66b antibody (clone G10F5, from novusbio; NB100-77808) was used to stain the TMAs on the automated Bond-Stainer from Leica, Germany. Whole slide images (WSI) were generated using a digital slide scanner (NanoZoomer S360, Hamamatsu Photonics) and expression of CD66b in the cytoplasm of polymorph neutrophils was assessed using Qupath (0.12), an open source software for digital pathology image analysis [18]. Qupath allows the detection and analysis of stained cells in brightfield images of individual TMA spots, as described previously in detail. [19, 20] In brief, the primary analysis included the TMA dearrayer to detect each single TMA spot. For optimal detection, the dearrayer was adjusted to each individual TMA spot. After that, color deconvolution was performed to separate the stains, which allows cell detection and analysis in conventional brightfield images using a watershed algorithm [21].

To identify the background intensity and the stained vectors based on the colors which are present in the WSI, the stain vectors were estimated. The 3,3’-Diaminobenzidine (DAB) thresholds were equally set within the evaluated groups to avoid false counts due to background staining and positive cells were detected for each spot. Finally, the positive cells were checked for plausibility of detection by a human observer (online resource 1).

### Statistical analysis

Patient data were prospectively collected. Interdependences between staining, tumor characteristics and clinical data were compared using the Pearson´s chi-squared test and Fisher´s exact test, illustrated by cross-tables. Overall survival was evaluated from the date of surgery until death. Kaplan–Meier curves were generated and compared using a log-rank test. Patient data with no events or lost follow up were censored at the last known date. Multivariate analysis for prognostic factors was performed using a Cox regression model. Included were factors that could potentially affect survival. ENTER was used here, since this method inserts all variables into the model at the same time. A two-sided *p* value < 0.05 was considered as statistically significant. SPSS package version 25 (IBM, Armonk, New York) was used for all statistical analyses.

## Results

### First cohort: gastric adenocarcinoma (*n* = 458)

Within the gastric carcinoma cohort we were able to include the results of the CD66b analyses from 458 patients.

With regard to sex the majority of patients were male (67,7%) and over 50 years of age (86,6%). Advanced UICC stages 3 and 4 were documented in 49% of cases. Proximal located carcinomas were more frequent (41,6%) than distal carcinomas (22,1%). According to the four molecular subtypes defined by the TCGA, the majority of tumors belonged to the CIN subgroup (73,5%), followed by GS tumors (13,1%) MSI tumors (8,8%) and EBV-positive tumors (4,5%). We then divided this cohort into (1) primarily resected gastric carcinomas and (2) neoadjuvant-treated tumors:

- (1) For all 268 patients within the first sub-cohort of the primarily resected gastric carcinoma the CD66b status could be determined. This subgroup showed a similar distribution regarding sex, age, UICC stage and localization. There were slightly more MSI- and GS subtypes represented (Tables 1, 2 and 3).
- (2) The second sub-cohort of neoadjuvant-treated gastric carcinomas (*n* = 190) the CD66b status of all patients could be determined. Again, this subgroup showed a similar distribution with regard to sex, age, UICC stage, TCGA subtypes and localization (lower proportion of distal tumors at 15.5%) (Tables 1, 2 and 3).

**Table 2:** Patient characteristics of the gastric adenocarcinoma collective separated into the sex, CIN- cohort and Non-CIN cohort

	CIN cohort (*n* = 308)				Non-CIN cohort (*n* = 111)			
	Male (*n* = 208)		Female (*n* = 100)		Male (*n* = 72)		Female (*n* = 39)	
Preoperative treatment								
None	120	57.7%	60	60%	44	61.1%	26	66.7%
Neoadjuvant	88	42.3%	40	40%	28	38.9%	13	33.3%
Age								
< 50	21	10.6%	17	18.5%	7	9.7%	7	18.9%
> 50	177	89.4%	75	81.5%	65	90.3%	30	81.1%
UICC stage								
(y)1	43	23.8%	21	24.4%	13	19.1%	8	22.9%
(y)2	52	28.7%	30	34.9%	16	23.5%	7	20%
(y)3	61	33.7%	20	23.3%	27	39.7%	16	45.7%
(y)4	25	13.8%	15	17.4%	12	17.6%	4	11.4%
Localisation								
Proximal	95	47%	32	32%	37	52.9%	9	23.7%
Corpus	42	20.8%	42	42%	15	21.4%	13	34.1%
Distal	40	19.8%	24	24%	15	21.4%	12	31.6%
Other	25	12.4%	2	2%	3	4.3%	4	10.5%

*TCGA* The Cancer Genome Atlas, *UICC* Union internationale contre le cancer, *CIN* chromosomal instability

**Table 3:** Distribution of CD66b + TANs in gastric carcinoma

	Amount CD66b + TAN in overall collective (*n* = 458)		Amount CD66b + TAN in primary surgery cohort (*n* = 268)		Amount CD66b + TAN in neoadjuvant cohort (*n* = 190)	
	Low	High	Low	High	Low	High
Sex						
Male	150 (48.4%)	160 (51.6%)	82 (46.6%)	94 (53.4%)	68 (50.7%)	66 (49.3%)
Female	80 (54.1%)	68 (45.9%)	49 (53.3%)	43 (46.7%)	31 (55.4%)	25 (44.6%)
Age						
< 50	30 (51.7%)	28 (48.3%)	13 (54.2%)	11 (45.8%)	17 (50.0%)	17 (50.0%)
> 50	187 (49.7%)	189 (50.3%)	115 (48.3%)	123 (51.7%)	72 (52.2%)	66 (47.8%)
UICC stage						
(y)1	25 (27.2%)	67 (72.8%)	13 (22.8%)	44 (77.2%)	12 (34.3%)	23 (65.7%)
(y)2	64 (56.1%)	50 (43.9%)	41 (55.4%)	33 (44.6%)	23 (57.5%)	17 (42.5%)
(y)3	74 (54.0%)	63 (46.0%)	44 (58.7%)	31 (41.3%)	30 (48.4%)	32 (51.6%)
(y)4	43 (70.5%)	18 (29.5%)	24 (63.2%)	14 (36.8%)	19 (82.6%)	4 (17.4%)
Molecular subtype						
CIN	155 (50.3%)	153 (49.7%)	86 (47.8%)	94 (52.2%)	69 (53.9%)	59 (46.1%)
MSI	6 (16.2%)	31 (83.8%)	4 (15.4%)	22 (84.6%)	2 (18.2%)	9 (81.8%)
GS	31 (56.4%)	24 (43.6%)	23 (63.9%)	13 (36.1%)	8 (42.1%)	11 (57.9%)
EBV	10 (52.6%)	9 (47.4%)	5 (62.5%)	3 (37.5%)	5 (45.5%)	6 (54.5%)
Localisation						
Proximal	90 (48.4%)	96 (51.6%)	50 (51.5%)	47 (48.5%)	40 (44.9%)	49 (55.1%)
Corpus	64 (51.2%)	61 (48.8%)	33 (47.1%)	37 (52.9%)	31 (56.4%)	24 (43.6%)
Distal	51 (51.5%)	48 (48.5%)	36 (50.7%)	35 (49.3%)	15 (53.6%)	13 (46.4%)
Other	19 (51.4%)	18 (48.6%)	12 (42.9%)	16 (57.1%)	7 (77.8%)	2 (22.2%)

*UICC* Union internationale contre le cancer, *CIN* chromosomal instability, *MSI* microsatellite instability, *GS* genomically stable, *EBV* Epstein–Barr virus-positive

### Gastric carcinoma cohort: TANs and prognosis

**Fig. 2:**
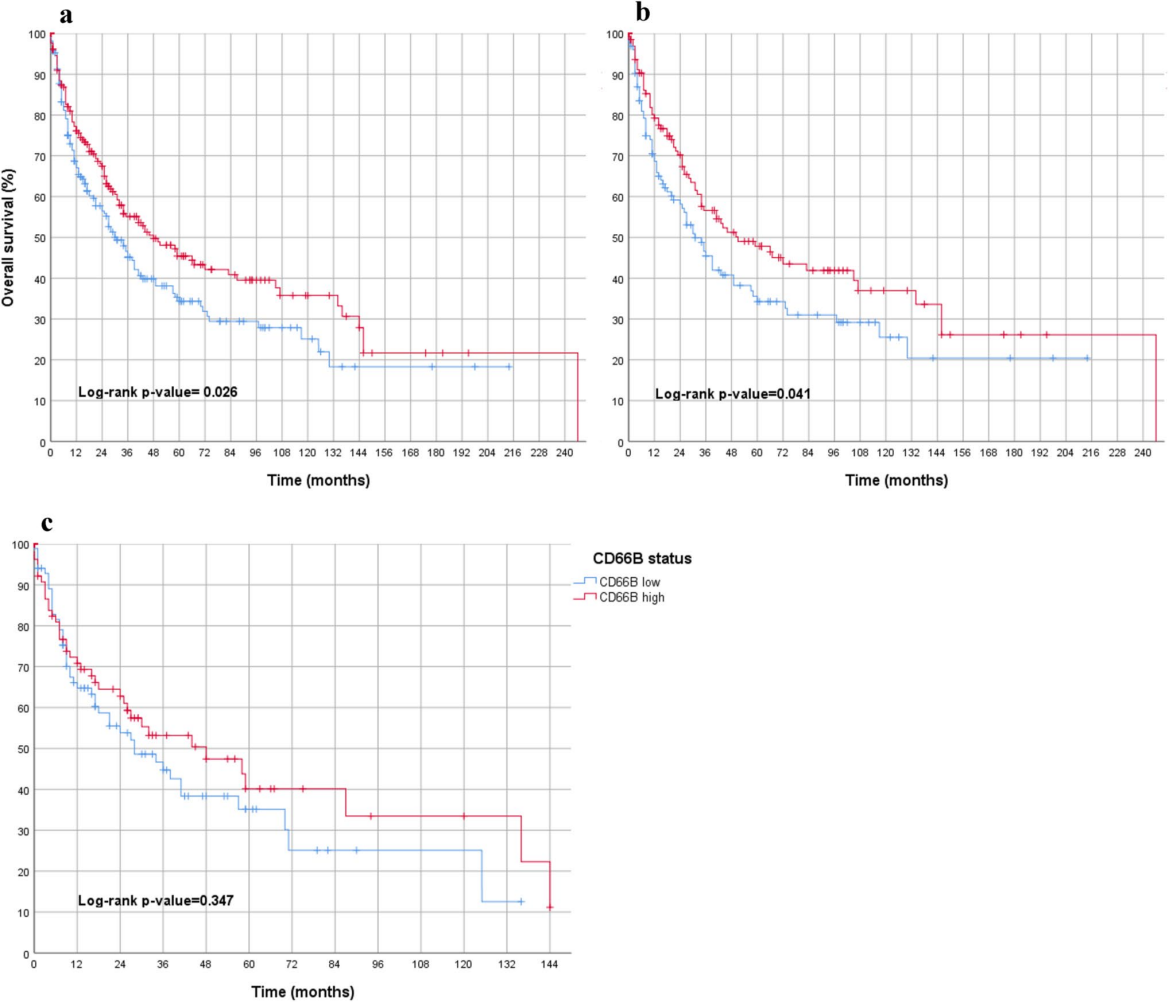
Overall survival analyses in the entire gastric carcinoma cohort (considering all molecular subtypes) in association to TANs in tumor stroma (blue = low density of TANs; red = high density of TANs) featuring **a** gastric carcinoma of all molecular subtypes including male and female patients, primary operated and neoadjuvant treated, showing a statistically significant survival advantage for CD66 high cases. **b** only primary operated gastric carcinoma of all molecular subtypes including male and female patients, showing a statistically significant survival advantage for CD66 high cases. **c** only neoadjuvant-treated gastric carcinoma of all molecular subtypes including male and female patients indicating no significant difference in survival between CD66 low and CD66 high patients

A high number of CD66b-positive TANs in tumor tissue was significantly associated with an improved overall survival (*p* = 0.026). The favorable prognostic effect on overall survival was also observed in the first subgroup of primarily resected patients (*p* = 0.041) but not in the second subgroup of neoadjuvant-treated patients (*p* = 0.347) (Fig. 2).

### Gastric carcinoma cohort: molecular subtypes and prognosis

**Fig. 3:**
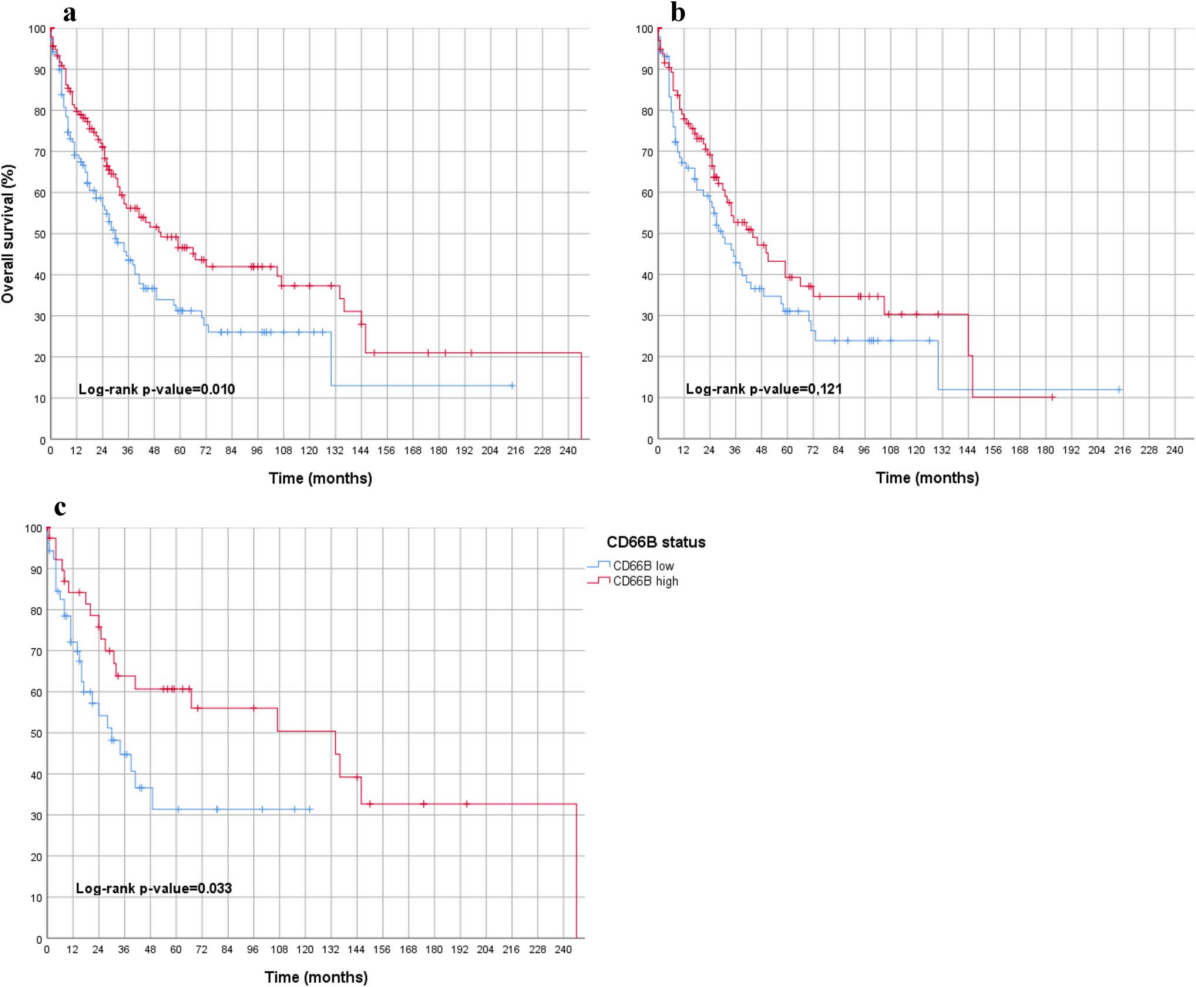
Overall survival analyses in CIN subgroup of gastric carcinoma in association to TANs in tumor stroma (blue = low density of TANs; red = high density of TANs) featuring **a** overall collective in the molecular CIN subtype of gastric carcinoma (including male, female, primarily resected and neoadjuvant treated), showing a statistically significant survival advantage for CD66 high cases. **b** only males: (primarily operated and neoadjuvant treated) gastric adenocarcinoma, CIN subtype, indicating no significant difference in survival between CD66 low and CD66 high patients. **c** only females (primarily operated and neoadjuvant treated) gastric adenocarcinoma, CIN subtype, showing a statistically significant survival advantage for CD66 high cases. *CIN* Chromosomal instability

The CIN tumors showed a positive prognostic association with a high number of TANs in the tumor tissue (*p* = 0.010) (Fig. 3). This was also observed in the subgroup of primarily resected tumors (*p* = 0.049), but not in the subgroup of neoadjuvant-treated CIN tumors (*p* = 0.092).

EBV-positive carcinomas, which had fewer CD66b-positive TANs, showed a favorable prognosis (*p* = 0.047). This association to prognosis within the EBV-positive tumors was lost in the two sub-cohorts (primary resected and neoadjuvant treated).

MSI tumors were significantly more frequent TAN-rich tumors. 81% of the MSI carcinomas harbored a higher number of CD66B-TANs compared to the median (*p* < 0.001). Within the group of MSI tumors, TANs had no prognostic significance. In comparison between TAN-rich MSI tumors and TAN-poor MSI carcinomas no difference in overall survival could be observed (*p* = 0.903).

For the GS tumors, no significant prognostic correlations with the amount of TANs in the tumor tissue was observed.

We presented the overall-survival curves of the non-CIN tumor subgroups separately by sex in the online supplement (online resource 3).

### Gastric carcinoma cohort: sex and prognosis

**Fig. 4:**
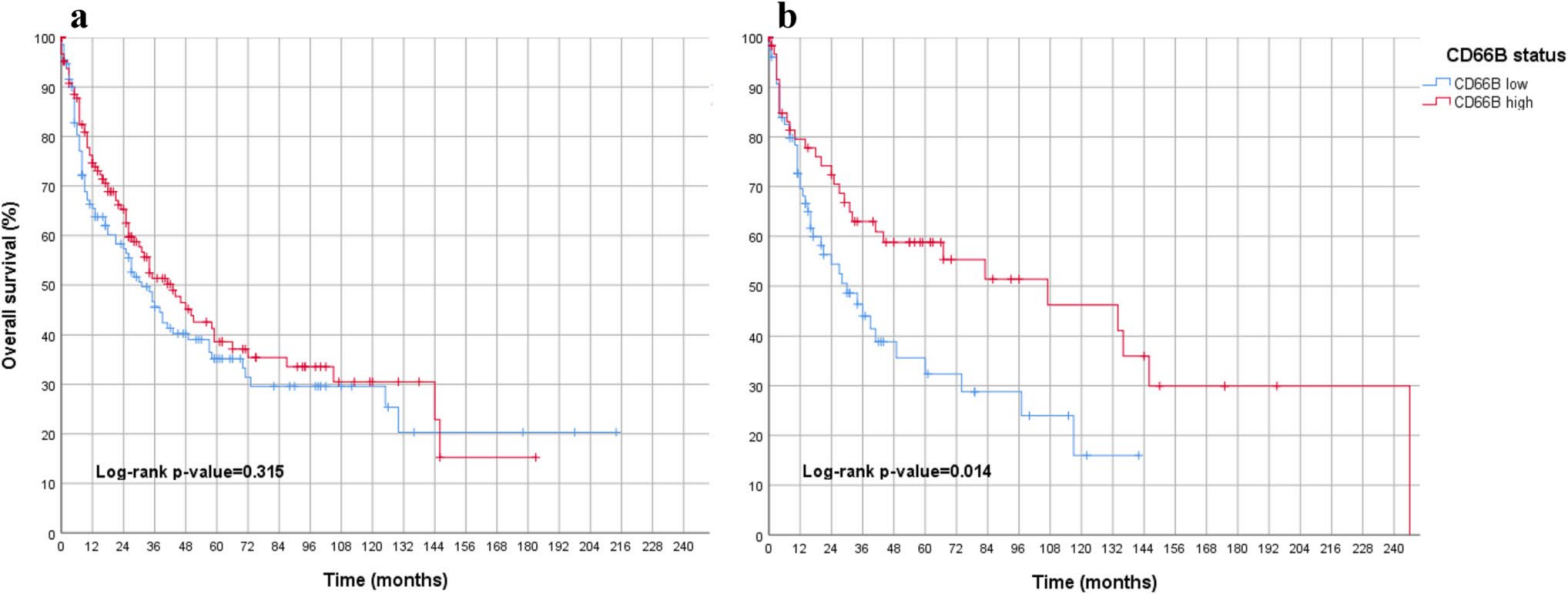
Overall survival analyses in the entire gastric carcinoma collective (primarily operated and neoadjuvant treated) separated into males and females in association to TANs in tumor stroma (blue = low density of TANs; red = high density of TANs) featuring **a** only males: primarily operated and neoadjuvant treated, indicating no significant difference in survival between CD66 low and CD66 high patients. **b** only females: primarily operated and neoadjuvant treated, showing a statistically significant survival advantage for CD66 high cases

The breakdown by sex revealed that the favorable prognosis was exclusively limited to female patients (Fig. 4).

In females, a significant positive correlation was found between high number of TANs and prognosis. This was observed in the overall cohort (*p* = 0.014, Fig. 4) and in the primarily surgical resected female individuals (*p* = 0.020). Conversely, no correlation was found in the subgroup of neoadjuvant-treated female individuals in the gastric tumor collective (*p* = 0.392).

To further prove the prognostic effect of the number of TANs in tumor tissue, we analyzed the effect with different cut-off values for the TANs (40, 50, 60th percentiles). Again, the TANs remained significantly associated with a better prognosis in female patients (in the 40th percentile, *p* = 0.015; 50th percentile, *p* = 0.014; 60th percentile, *p* = 0.025). Also, within the subgroup analysis of female patients with CIN tumors, TAN-rich tumors remained statistically significantly associated with better overall survival (*p* = 0.033; Fig. 3).

If only male patients are included in the various groups of the gastric cohort, the beneficial prognostic effect of TANs in tumor stroma, as previously demonstrated when considering all patients, was lost. There was no evidence for a prognostic relevance of CD66b-positive neutrophil granulocytes in the tumor tissue in the male total gastric cohort (*p* = 0.315) (Fig. 4), in the group of primarily surgical resected men (*p* = 0.417) or in men treated with neoadjuvant therapy (*p* = 0.550). In addition, within a subgroup analysis of male patients with chromosomally instable gastric carcinoma (CIN), TANs showed no prognostic significance (*p* = 0.121, Fig. 3).

Together, the molecular- and sex-specific subgroup analyses of the gastric carcinoma cohort showed that the favorable prognosis in tumors with a large amount of TANs, when the unfiltered, entire cohort is considered, is borne exclusively by female patients and the specific molecular subgroup of TAN-rich CIN tumors.

### Gastric carcinoma cohort: TANs and prognosis using multivariate analyses

There are significantly more neutrophils (TAN) in UICC stage 1 as well as in the microsatellite-instable subtype (MSI) (*p* =  < 0.001). This applies to both parameters for the total group as well as for the primarily operated group. In UICC stage 4, neutrophil-granulocyte-rich tumors are significantly less frequent (*p* = 0.003). Multivariate analyses were conducted to determine whether TANs were also an independent prognostic factor. The factors sex, age, molecular subtypes, neoadjuvant therapy, residual stage and aspects of the TNM stage (Table 4) were included in analyses. Here, it was found that the amount of TANs does not qualify as an independent prognostic value. This is of particular interest, as tumors that are non-nodally metastasised are mainly tan-rich ((y)pN0: 43.7% TAN-poor vs. 56.3% TAN-rich and -pN + : 53.1% TAN-poor vs. 46.9% TAN-rich (*p* = 0.042) (compare correlation co-efficient in online resource 2).

**Table 4:** Multivariate analysis of the gastric cancer collective

		(a) Whole cohort				(b) Female sex only			
		HR	95.0% CI for HR		*p* value	HR	95.0% CI for HR		*p* value
			Lower	Upper			Lower	Upper	
CD66b	Low	0.927	0.679	1.266	0.635	0.833	0.453	1.530	0.556
	High				Ref				Ref
UICC	Overall				0.000				0.000
	UICC 1	0.104	0.061	0.176	0.000	0.050	0.016	0.154	0.000
	UICC 2	0.137	0.085	0.220	0.000	0.124	0.051	0.298	0.000
	UICC 3	0.395	0.261	0.599	0.000	0.384	0.176	0.839	0.016
	UICC 4				Ref				Ref
Age	< 50 years	0.622	0.383	1.010	0.055	0.530	0.260	1.083	0.082
	> 50 years				Ref				Ref
Sex	Male	1.108	0.801	1.533	0.535				
	Female				Ref				
Treatment	Primary surgery	0.817	0.593	1.125	0.215	0.804	0.442	1.462	0.474
	Neoadjuvant treatment				Ref				Ref
TCGA	Overall				0.653				0.824
	CIN				Ref				Ref
	GS	0.968	0.620	1.512	0.886	1.316	0.628	2.758	0.467
	MSI	0.700	0.404	1.212	0.203	0.734	0.218	2.478	0.619
	EBV	0.942	0.482	1.842	0.862	0.929	0.199	4.341	0.926

*CI* Confidence interval, *HR* Hazard ratio, *TCGA* The Cancer Genome Atlas, *UICC* Union internationale contre le cancer, *CIN* chromosomal instability, *MSI* microsatellite instability, *GS* genomically stable, *EBV* Epstein–Barr virus-positive

### Esophageal adenocarcinoma cohort (*n* = 660)

Having shown a prognostic relevance of TANs within gastric adenocarcinomas, we used adenocarcinomas of the esophagus because they correspond to the molecular CIN subtype of the stomach.

As expected for adenocarcinomas of the esophagus, our cohort includes predominantly male patients (*n* = 578 (87.6%)). Advanced tumor stages ((y)UICC stages 3 and 4) are represented in nearly half of the cases (54.2%) (Table 5).

**Table 5:** Patient characteristics of the esophageal adenocarcinoma cohort

	Overall collective (*n* = 660)		Female (*n* = 82)		Male (*n* = 578)	
CD66b						
Low	333	50.5%	40	48.8%	293	50.7%
High	327	49.5%	42	51.2%	285	49.3%
Missing data	0		0		0	
Age						
< 65	336	52.7%	38	50.0%	298	53.1%
> 65	301	47.3%	38	50.0%	263	46.9%
Missing data	23	3.5%	6	7.3%	17	2.9%
pT stage						
pT1	97	14.8%	9	11.1%	88	15.3%
pT2	85	13.0%	11	13.6%	74	12.9%
pT3	450	68.6%	54	66.7%	396	68.9%
pT4	22	3.4%	6	7.4%	16	2.8%
Missing data	6	0.9%	2	2.4%	4	0.7%
Lymph node metastasis						
pN0	265	40.5%	33	40.2%	232	40.5%
pN1	219	33.4%	21	25.6%	198	34.6%
pN2	87	13.3%	14	17.1%	73	12.7%
pN3	84	12.8%	14	17.1%	70	12.2%
Missing data	5	0.8%	0	0.0%	5	0.9%
UICC stage						
(y)1	139	21.3%	15	18.8%	124	21.6%
(y)2	160	24.5%	20	25.0%	140	24.4%
(y)3	276	42.3%	34	42.5%	242	42.2%
(y)4	78	11.9%	11	13.8%	67	11.7%
Missing data	7	1.1%	2	2.4%	5	0.9%

*UICC* Union internationale contre le cancer

We then divided this second cohort similarly to the first cohort into (1) primarily resected esophageal tumors and (2) neoadjuvant-treated tumors:

(1) The first sub-cohort with primarily resected esophageal adenocarcinoma included 275 patients and the second subgroup (2) of neoadjuvant-treated esophageal adenocarcinoma 385 patients. Both groups showed similar distributions regarding sex, age, UICC stage (lower proportion of UICC stages 3–4 in the first subgroup).

### TANs and prognosis

**Fig. 5:**
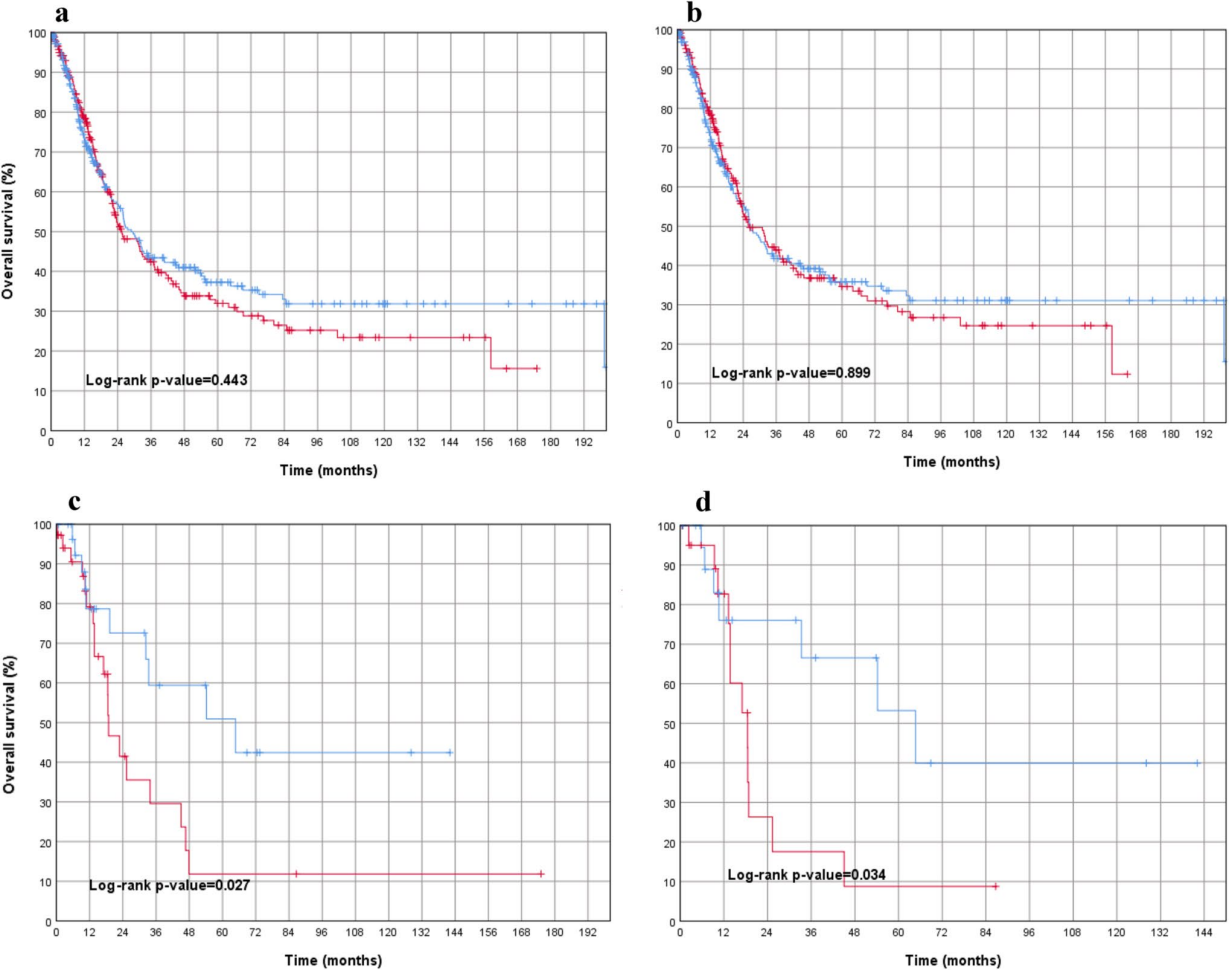
Overall survival analyses in esophageal adenocarcinoma. (red = low density of TANs; blue = high density of TANs) featuring **a** overall esophageal adenocarcinoma (including males, females, primarily operated and neoadjuvantly treated adenocarcinomas of the esophagus), indicating no significant difference in survival between CD66 low and CD66 high patients. **b** males (primarily operated and neoadjuvant treated) with adenocarcinoma of the esophagus, indicating no significant difference in survival between CD66 low and CD66 high patients. **c** females (primarily operated and neoadjuvant treated) with adenocarcinoma of the esophagus, showing a statistically significant survival advantage for CD66 high cases. **d** females with neoadjuvant-treated adenocarcinomas of the esophagus, showing a statistically significant survival advantage for CD66 high cases

The distribution of CD66b + TANs in esophageal adenocarcinoma is shown in Table 6**.** When considering all tumors in this cohort (*n* = 660), no statistically measurable effect was found with regard to prognosis (*p* = 0.443). This was confirmed when looking at male patients only (*p* = 0.899, Fig. 5).

**Table 6:** Distribution of CD66b + TANs in esophageal adenocarcinoma

	Amount CD66b + TAN in overall collective (*n* = 660)		Amount CD66b + TAN in primary surgery cohort (*n* = 275)		Amount CD66b + TAN in neoadjuvant cohort (*n* = 385)	
	Low	High	Low	High	Low	High
Sex						
Male	281 (48.6%)	297 (51.4%)	121 (49.6%)	123 (50.4%)	160 (47.9%)	174 (52.1%)
Female	43 (52.4%)	39 (47.6%)	18 (58.1%)	13 (41.9%)	25 (49.0%)	26 (51.0%)
Age						
< 65	155 (46.3%)	180 (53.7%)	54 (47.0%)	61 (53.0%)	101 (45.9%)	119 (54.1%)
> 65	150 (50.8%)	145 (49.2%)	71 (50.0%)	71 (50.0%)	79 (51.6%)	74 (48.4%)
UICC stage						
(y)1	54 (38.8%)	85 (61.2%)	30 (38.0%)	49 (62.0%)	24 (40.0%)	36 (60.0%)
(y)2	77 (48.1%)	83 (51.9%)	29 (47.5%)	32 (52.5%)	48 (48.5%)	51 (51.5%)
(y)3	140 (50.7%)	136 (49.3%)	56 (58.9%)	39 (41.1%)	84 (46.4%)	97 (53.6%)
(y)4	48 (61.5%)	30 (38.5%)	21 (60.0%)	14 (40.0%)	27 (62.8%)	16 (37.2%)

*UICC* Union internationale contre le cancer

Interestingly, if only female patients were analyzed (primarily resected and neoadjuvantly treated patients), a highly statistically significant association to overall survival was found again in female patients whose stroma was rich in TANs (*p* = 0.027) (Fig. 5). The primarily resected esophageal carcinomas (*n* = 275) showed no prognosis association when both sexes are considered (*p* = 0.865). This applied to male patients with primary resected esophageal cancer (*p* = 0.682) and female patients with primary surgery (*p* = 0.518), although only 31 women were represented in this subgroup. A statistical significance for TAN-rich carcinomas was found in both, the total group of female patients and in those treated with neoadjuvant therapy.

(2) The neoadjuvant-treated esophageal carcinomas (*n* = 385) again showed no prognostic association of TANs when both sexes were considered (*p* = 0.240), or when male patients were analyzed as a subgroup (*p* = 0.627). In contrast, the isolated analysis of neoadjuvant treated and secondarily resected female patients (*n* = 46) showed a significantly better overall survival when the tumor stroma was enriched with TANs (*p* = 0.034) (Fig. 5).

In summary, the results of the esophageal cohort confirmed the results of the gastric tumor cohort. TANs were relevant for prognosis in specific subgroups of adenocarcinomas of the stomach and the esophagus. Female individuals with gastric adenocarcinoma showed an improved overall survival if their tumors harbored a TAN-rich stroma. This was particularly evident in the molecular subtype of chromosomally instable (CIN) tumors. This effect was not observed in male patients. In accordance with the comparable molecular characteristics, we were also able to demonstrate this biological relevance in the small group of female patients with adenocarcinoma of the esophagus.

## Discussion

The importance of tumor associated neutrophils (TAN) in solid tumors remains a topic of current interest. In a recent study, Zhao et al. found an association of unfavorable prognosis within a collection of Asian individuals (*n* = 212) by assessing CD15 + TANs within the tumor stroma of gastric cancer cases. Of importance, CD15 is also expressed by other inflammatory cells, like granulocytes, monocytes, as well as eosinophils in addition to neutrophils. [22] For this reason, Zhang et al. have analyzed a collection of gastric cancer cases (*n* = 305) using a more or less TAN-specific CD66b antibody. [9] Here, it was found that TANs were associated to a favorable prognosis. Another study by Caruso et al. described a favorable prognosis of TANs using a cohort from South Europe patient collective of gastric carcinomas (*n* = 273). The neutrophils in the stroma were counted standard morphologically in this study. [23] Therefore, we decided to use a CD66b-detecting antibody but in parallel to this, a pathologist (BSM) with many years of experience determined the distribution of polymorph neutrophils in tumor tissue by standard morphology. In the HE-stained section, neutrophils can be easily distinguished from eosinophilic granulocytes, some of which are also labeled with CD66b. The semi-quantitative ("human") results were also statistically evaluated (data not shown). With this semi-quantitative determination we also achieved comparable statistical results. We have decided to present only the software-measured results for publication, because they allow a higher degree of reproducibility and the technique was already used in a previous work.

Recently, a study analyzing 449 Caucasian patients with adenocarcinomas of the stomach was also able to show the positive prognostic effect of TANs in the tumor stroma–limited to female sex. This study used a different immunohistochemical staining of myeloperoxidase (MPO) to visualize TANs. In accordance with our results, no prognostic relevance was found in males. The results of this work are very significant, since the same sex-specific phenomenon of TANs is described in two different tumor collectives, which underlines the probability of potential underlying biological relevance. [24].

Currently, the association to the corresponding molecular subtypes of gastric cancer and TANs remain elusive, although the WHO classification of tumors (2019) highlights its clinical relevance. Interestingly, our analysis of the molecular subgroups revealed that this favorable prognosis was related to the molecular CIN subtype. As adenocarcinoma of the esophagus have almost exclusively the same molecular characteristics as the CIN subtype of adenocarcinomas of the stomach, we included these cases to our analysis as well. As of now, there is a lack of data for adenocarcinoma of the esophagus and TANs. Interestingly, the results of the overall cohort of esophageal carcinomas showed no prognostic relevance of TANs. Although, a sub-analysis illustrated a sex-specific prognostic relevance for TANs in female individuals. Typically for the demographic aspect of esophageal adenocarcinoma, the cohort mainly represents individuals of male sex (90%). By looking into the subgroup of female patients with adenocarcinoma of the esophagus, we could confirm the favorable prognostic effect of TANs in the tumor stroma. The multivariate analysis considering the UICC stages showed that TAN-rich tumors were not an independent prognostic factor, since TAN-rich tumors were particularly enriched in lympho-nodal-negative tumors.

Of particular interest to our study, sex-specific differences in prognosis or treatment response in solid tumors have come into scientific focus. [25] Several studies showed sex-related differences in the functionality and responsiveness of neutrophils. For example, it has been shown that progesterone inhibits the apoptosis tendency of neutrophils and thus mediating a longer neutrophil lifespan. [26, 27] It remains to be determined, which hormonal changes were relevant in our predominantly older, postmenopausal patient population of female individuals.

Today, a large proportion of gastric, as well as esophageal cancers receive neoadjuvant (radio-)chemotherapy. The effects of pre-surgical therapy on the composition of TANs in the tumor stroma have not been considered so far. The favorable prognosis that we–and others–see in primarily resected gastric carcinomas is lost within a neoadjuvant setting in female patients. Interestingly, this is not the case with neoadjuvantly treated adenocarcinoma of the esophagus. Even after neoadjuvant treatment, the TAN-rich tumor stroma was associated to a favorable prognosis in female patients (*p* = 0.034). Cytostatic drugs may alter the composition of the local inflammatory tumor microenvironment. We have recently described this effect in esophageal adenocarcinoma. [28] To what extent additional radiotherapy has a "more beneficial" effect on this local inflammatory stroma with regard to the TAN composition remains unknown.

As of now, the underlying patho-mechanism of TANs in the stroma of human tumors remains unclear. At least in mouse models, two different TANs are described- TAN1 with tumor-inhibiting properties and TAN2 with tumor-promoting properties. Which subtype predominates in the tumor stroma is presumably related to the composition of specific cytokines. Here, neutrophils migrate from the blood into the tumor stroma and become tumor-inhibiting TAN1 if an interferon-ß (INFß)-rich and tumor growth factor-ß (TNFß)-poor microenvironment is present. Neutrophils may inhibit local tumor growth or affect the ability of tumor cells to metastasize, inter alia, by direct cytotoxicity (via oxygen radicals) or by trail-mediated apoptosis enhancement in tumor cells. [7] In contrast, a TNFß-rich tumor micromileu leads to an accumulation of tumor-promoting TANs. Mechanistically, the cytokine composition within the tumor stroma is multifactorial and, among other things, determined by the mutation properties of the given tumor. They succeed in doing so by means of various cytokines (e.g. activation of *K-RAS* leads to an accumulation of TANs with tumor-promoting properties). The results presented here for the CIN subtype of gastric carcinoma are also consistent with this. In addition, microsatellite-instable gastric carcinomas in our cohort show an accumulation of TANs in the stroma (in 83.8%). The MSI subgroup of gastric carcinomas (which is almost non-existent in the esophagus) is associated with a more favorable prognosis overall, which cannot be further increased by TANs (10, 29). Methodological weaknesses of this work are its retrospective character and the consideration of a solitary tumor center. As mentioned above, another study has found an identical sex aspect with polymorph neutrophils in gastric carcinoma, thus relativizing the problem of the solitary tumor center, since two independent cohorts came to similar conclusions. It is useful to verify our results in a prospective clinical study. This should be technically and methodologically easy to realize.

The pathophysiological mechanism that can explain this sex-specific difference must, however, also be left out of this manuscript. We could only speculate at this stage. Since mouse models have clear limitations (as mentioned above), a promising first approach would be quantification of patient blood and FACS analysis of tumor tissue, focusing on the various immunological aspects. This could be combined with molecular tumor analysis by whole-genome sequencing. Possibly, this could provide evidence for sex-specific differences of the antigen presentation machinery by tumor cells or specific interleukin patterns.

In summary, our study reveals that TANs show a sex-specific prognostic effect in female individuals. This applies to adenocarcinomas of the stomach–with an emphasis on the CIN subgroup of gastric adenocarcinomas–and also to the related adenocarcinoma of the esophagus. Future studies dealing with the significance of TANs in malignant tumors may appreciate the relevance of the observed differences between female and male sex in our study. In addition, the prognostic and possible predictive significance of TANs in prospective studies must consider pre-therapeutic interventions which may affect this as a potential biomarker.

## Supplementary Information

Below is the link to the electronic supplementary material.

Histology and digital measurement of CD66b positive polymorph neutrophils (PDF 8352 KB)

Correlation co-efficient in TAN-rich gastric carcinoma (PDF 41 KB)

Overall survival analyses in gastric carcinoma of the different molecular subtypes according to sex: a) Non-CIN gastric tumors in males b) Non-CIN gastric tumors in females (PDF 509 KB)
