# Supplementary material for: Sex-specific prognostic effect of CD66b-positive tumor-infiltrating neutrophils (TANs) in gastric and esophageal adenocarcinoma
Source: Gastric Cancer. 2021 May 19;24(6):1213–26. doi: 10.1007/s10120-021-01197-2 (PMC8502159; doi:10.1007/s10120-021-01197-2)
Supplement: Supplementary file 2 — Correlation co-efficient in TAN-rich gastric carcinoma (PDF 41 KB) [file 10120_2021_1197_MOESM2_ESM.pdf]

Sex specific prognostic effect of CD66b-positive Tumor-infiltrating neutrophils (TANs) in gastric and esophageal adenocarcinoma

Gastric Cancer

Alexander Quaas[2#], Aylin Pamuk[1#], Sebastian Klein[2], Jennifer Quantius[2], Jan Rehkaemper[2], Atakan G. Barutcu[1], Josef Rueschoff[4], Thomas Zander[3], Florian Gebauer[1], Axel Hillmer[2], Reinhard Buettner[2], Wolfgang Schroeder[1], Christiane J. Bruns[1], Heike Löser[2], Birgid Schoemig-Markiefka[2#] and Hakan Alakus[1#]

1 Department of General, Visceral, Cancer and Transplantation Surgery, University Hospital Cologne, Cologne (Germany)
2 Institute of Pathology, University Hospital Cologne, Cologne (Germany)
3 University of Cologne, Department I of Internal Medicine, Center for Integrated Oncology Aachen Bonn Cologne Duesseldorf; Gastrointestinal Cancer Group Cologne GCGC
4 Institute of Pathology, Nordhessen and Targos Molecular Pathology GmbH, Kassel, (Germany)
# contributed equally to the work

Correspondence to
PD Dr med Hakan Alakus
Department of General, Visceral, Cancer and Transplantation Surgery
University Hospital Cologne
Kerpener Str. 62
50937 Cologne, Germany
E-Mail: hakan.alakus@uk-koeln.de
Phone: +49 0221 478-4803
Fax: +49 0221 478-6258

Online Resource 2 Correlation co-efficient in TAN-rich gastric carcinoma

			TAN rich (>50. percentiles)	(y)pN-Category
Spearman-Rho	TAN rich (> 50. percentiles)	correlation co-efficient	1,000	-,141**
		Sig. (2-sided)	.	,003
		N	556	439
	(y)pN-Category	correlation co-efficient	-,141**	1,000
		Sig. (2-sided)	,003	.
		N	439	439

**. The correlation is significant at the 0,01 level (two-sided).
