# Supplementary material for: Sex-specific prognostic effect of CD66b-positive tumor-infiltrating neutrophils (TANs) in gastric and esophageal adenocarcinoma
Source: Gastric Cancer. 2021 May 19;24(6):1213–26. doi: 10.1007/s10120-021-01197-2 (PMC8502159; doi:10.1007/s10120-021-01197-2)
Supplement: Supplementary file 3 — Overall survival analyses in gastric carcinoma of the different molecular subtypes according to sex: a) Non-CIN gastric tumors in males b) Non-CIN gastric tumors in females (PDF 509 KB) [file 10120_2021_1197_MOESM3_ESM.pdf]

Sex specific prognostic effect of CD66b-positive Tumor-infiltrating neutrophils (TANs) in gastric and esophageal adenocarcinoma

Gastric Cancer

Alexander Quaas[2#], Aylin Pamuk[1#], Sebastian Klein[2], Jennifer Quantius[2], Jan Rehkaemper[2], Atakan G. Barutcu[1], Josef Rueschoff[4], Thomas Zander[3], Florian Gebauer[1], Axel Hillmer[2], Reinhard Buettner[2], Wolfgang Schroeder[1], Christiane J. Bruns[1], Heike Löser[2], Birgid Schoemig-Markiefka[2#] and Hakan Alakus[1#]

1 Department of General, Visceral, Cancer and Transplantation Surgery, University Hospital Cologne, Cologne (Germany)
2 Institute of Pathology, University Hospital Cologne, Cologne (Germany)
3 University of Cologne, Department I of Internal Medicine, Center for Integrated Oncology Aachen Bonn Cologne Duesseldorf; Gastrointestinal Cancer Group Cologne GCGC
4 Institute of Pathology, Nordhessen and Targos Molecular Pathology GmbH, Kassel, (Germany)
# contributed equally to the work

Correspondence to
PD Dr med Hakan Alakus
Department of General, Visceral, Cancer and Transplantation Surgery
University Hospital Cologne
Kerpener Str. 62
50937 Cologne, Germany
E-Mail: hakan.alakus@uk-koeln.de
Phone: +49 0221 478-4803
Fax: +49 0221 478-6258

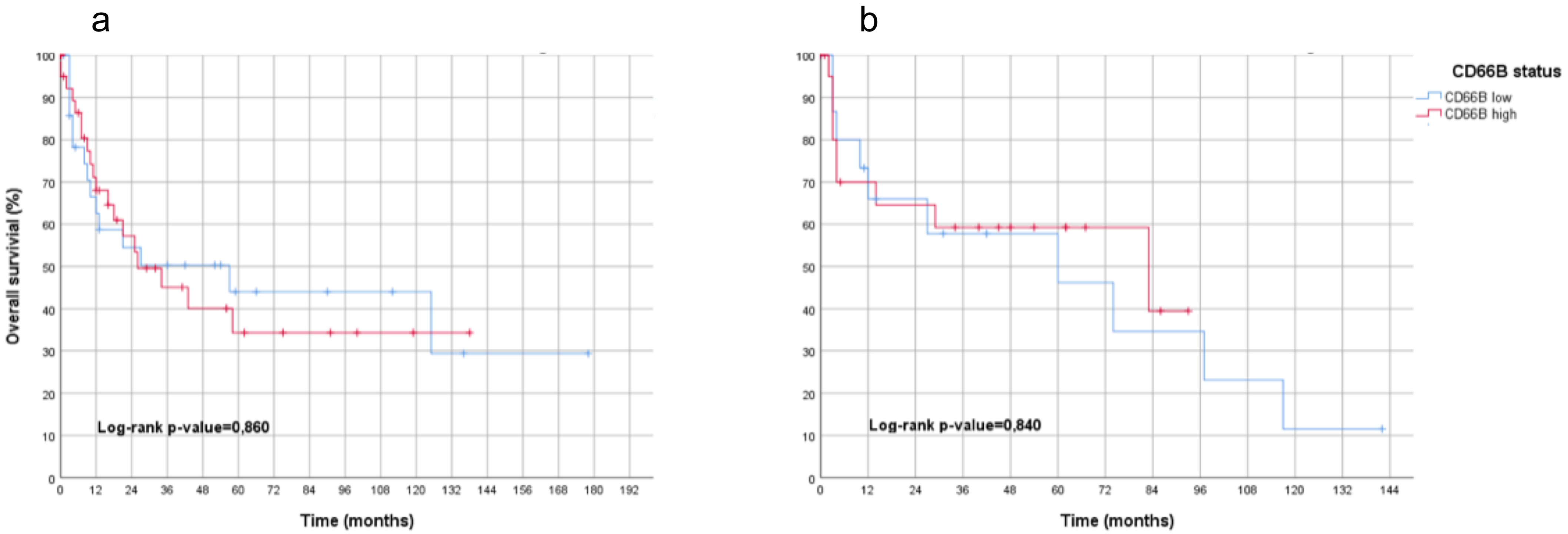

Online Resource 3 Overall survival analyses in gastric carcinoma of the different molecular subtypes according to sex (blue =low density of TANs; red = high density of TANs)

a) Non-CIN gastric tumors in males

b) Non-CIN gastric tumors in females
